# Supplementary material for: Chronic Viral Hepatitis Signifies the Association of Premixed Insulin Analogues with Liver Cancer Risks: A Nationwide Population-Based Study
Source: Int J Environ Res Public Health. 2019 Jun 13;16(12):2097. doi: 10.3390/ijerph16122097 (PMC6616640; doi:10.3390/ijerph16122097)
Supplement: Supplementary file 1 [file ijerph-16-02097-s001.pdf]

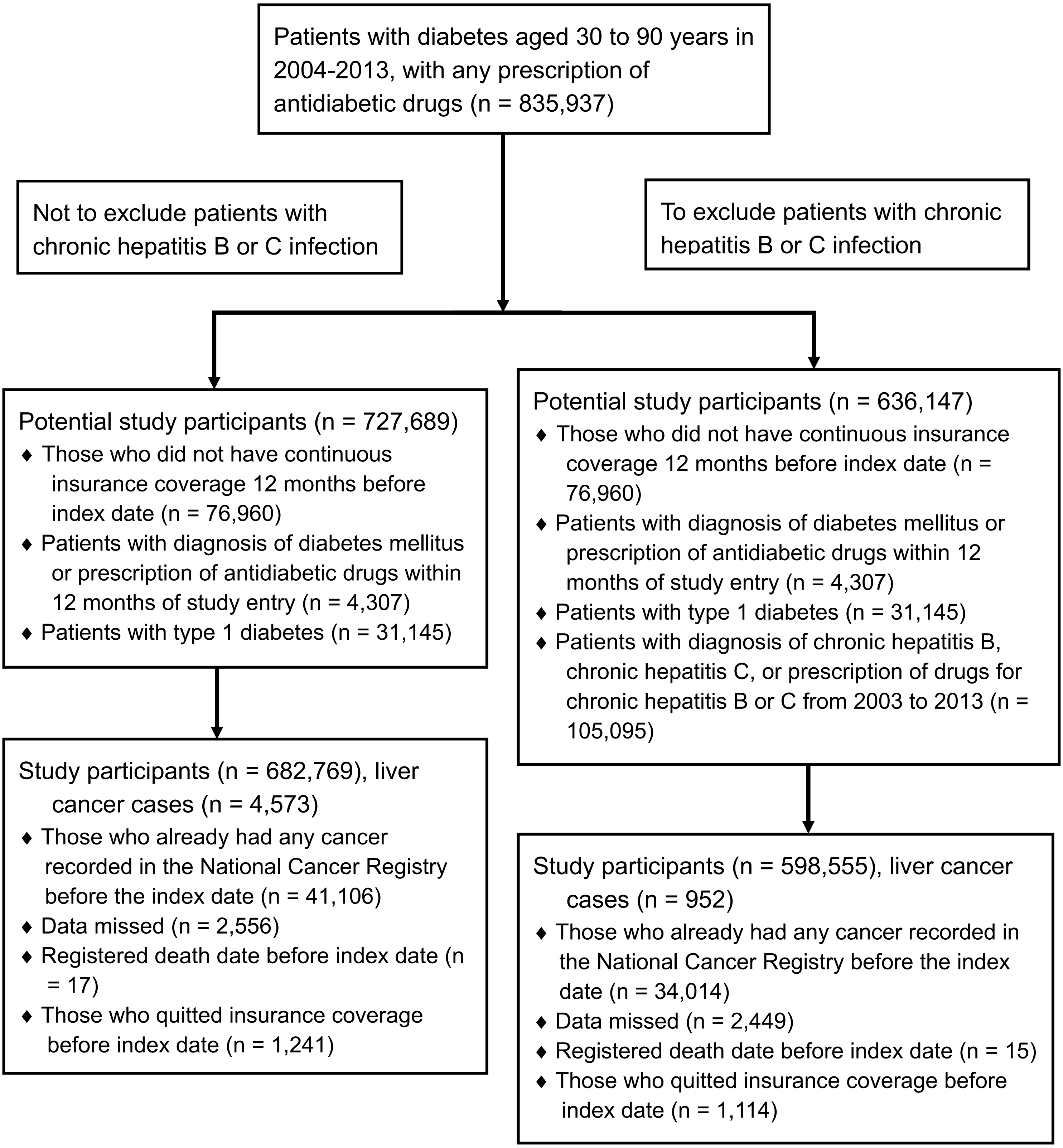

Figure S1

Study flowchart from 2004 to 2013 before and after exclusion of patients with chronic viral hepatitis

Table S1 Hepatocellular carcinoma cases and matched controls among newly diagnosed type 2 diabetes patients prescribed any antidiabetic agents (from 2003 to 2013)

	Cases (N=5,832)	Controls (N=23,328)	Crude OR (95% CI)
Age	60.9 (11.1)	60.7 (11.1)	-
Male	4,274 (73.3)	17,096 (73.3)	-
Year of initiating antidiabetic agents			
2003	793 (13.6)	3,172 (13.6)	-
2004	856 (14.7)	3,424 (14.7)	-
2005	787 (13.5)	3,148 (13.5)	-
2006	663 (11.4)	2,652 (11.4)	-
2007	739 (12.7)	2,956 (12.7)	-
2008	577 (9.9)	2,308 (9.9)	-
2009	555 (9.5)	2,220 (9.5)	-
2010	426 (7.3)	1,704 (7.3)	-
2011	272 (4.7)	1,088 (4.7)	-
2012	147 (2.5)	588 (2.5)	-
2013	17 (0.3)	68 (0.3)	-
Socioeconomic status (monthly income in NTD)			
≤17,280	2,043 (35.0)	9,445 (40.5)	Reference
17,281~22,800	2,467 (42.3)	8,492 (36.4)	1.35 (1.26-1.44)
22,801~28,800	290 (5.0)	1,217 (5.2)	1.11 (0.97-1.28)
28,801~36,300	328 (5.6)	1,155 (5.0)	1.32 (1.15-1.51)
36,301~45,800	438 (7.5)	1,570 (6.7)	1.30 (1.15-1.46)
>45,800	266 (4.6)	1,449 (6.2)	0.85 (0.74-0.99)
Medication use before cancer diagnosis			
Insulin analogues	589 (10.1)	1,188 (5.1)	2.11 (1.90-2.34)
Premixed insulin analogues	363 (6.2)	610 (2.6)	2.48 (2.17-2.84)
Oral antidiabetic drugs	5,113 (87.7)	21,416 (91.8)	0.63 (0.57-0.69)
ACEi/ARBs	396 (6.8)	1,745 (7.5)	0.90 (0.80-1.01)
Alpha-blockers	70 (1.2)	245 (1.1)	1.15 (0.88-1.50)
Beta-blockers	793 (13.6)	2,118 (9.1)	1.60 (1.47-1.75)
Calcium channel blockers	290 (5.0)	1,207 (5.2)	0.96 (0.84-1.10)
Dihydropyridine	278 (4.8)	1,170 (5.0)	0.95 (0.83-1.08)
Non-dihydropyridine	12 (0.2)	43 (0.2)	1.12 (0.59-2.12)
Diuretics	458 (7.9)	554 (2.4)	3.57 (3.14-4.07)
Statins	543 (9.3)	4,285 (18.4)	0.45 (0.41-0.49)
Fibrates	442 (7.6)	3,171 (13.6)	0.51 (0.46-0.57)
Comorbidities			

Liver cirrhosis	3,228 (55.4)	1,292 (5.5)	21.5 (19.6-23.6)
Chronic hepatitis B	2,929 (50.2)	1,060 (4.5)	21.8 (19.7-24.0)
Chronic hepatitis C	1,839 (31.5)	242 (1.0)	44.5 (37.9-52.3)
Hypertension	2,157 (37.0)	8,746 (37.5)	0.98 (0.92-1.04)
Hyperlipidemia	556 (9.5)	4,299 (18.4)	0.47 (0.42-0.51)
Ischemic heart disease	426 (7.3)	1,984 (8.5)	0.85 (0.76-0.94)
Myocardial infarction	40 (0.7)	220 (0.9)	0.73 (0.52-1.02)
Heart failure	179 (3.1)	575 (2.5)	1.26 (1.06-1.49)
Atrial fibrillation	76 (1.3)	261 (1.1)	1.17 (0.90-1.51)
Cerebrovascular disease	371 (6.4)	1,867 (8.0)	0.78 (0.69-0.87)
Stroke	243 (4.2)	1,193 (5.1)	0.80 (0.70-0.93)
Peripheral vascular disease	30 (0.5)	131 (0.6)	0.92 (0.62-1.36)
Chronic kidney disease	185 (3.2)	521 (2.2)	1.44 (1.21-1.70)
Depression	101 (1.7)	448 (1.9)	0.90 (0.72-1.12)
Charlson index	2.5 (2.4)	1.0 (1.2)	1.67 (1.64-1.70)
Resource utilization			
Number of A1C measurement	5.0 (6.2)	4.6 (6.0)	1.01 (1.01-1.02)
Number of lipid measurement	3.9 (4.9)	4.3 (5.4)	0.98 (0.98-0.99)
Number of outpatient visits	85.6 (92.2)	73.4 (83.5)	1.00 (1.00-1.00)
Number of hospitalization	1.8 (2.9)	1.0 (2.2)	1.14 (1.13-1.16)
Length of hospital stay > 7 days	0.9 (1.7)	0.5 (1.4)	1.17 (1.15-1.19)

Data presented as mean (standard deviation) or number (percent). Insulin analogues included insulin glargine, insulin detemir and premixed insulin analogues (insulin aspart plus insulin aspart protamine and insulin lispro plus insulin lispro protamine). ACEi, angiotensin-converting-enzyme inhibitor; ARB, angiotensin II receptor blocker.

Table S2 Hepatocellular carcinoma cases and matched controls among newly diagnosed type 2 diabetes patients prescribed any antidiabetic agents (from 2004 to 2013)

	Cases (n=4,573)	Controls (n=18,292)	Crude OR (95% CI)
Age	61.1 (11.1)	60.8 (11.1)	-
Male	3,384 (74.0)	13,536 (74.0)	-
Year of initiating antidiabetic agents			
2004	704 (15.4)	2,816 (15.4)	-
2005	708 (15.5)	2,832 (15.5)	-
2006	609 (13.3)	2,436 (13.3)	-
2007	675 (14.8)	2,700 (14.8)	-
2008	538 (11.8)	2,152 (11.8)	-
2009	515 (11.3)	2,060 (11.3)	-
2010	405 (8.9)	1,620 (8.9)	-
2011	260 (5.7)	1,040 (5.7)	-
2012	144 (3.2)	576 (3.2)	-
2013	15 (0.3)	60 (0.3)	-
Socioeconomic status (monthly income in NTD)			
≤17,280	1,578 (34.5)	7,500 (41.0)	Reference
17,281~22,800	1,934 (42.3)	6,507 (35.6)	1.42 (1.31-1.53)
22,801~28,800	230 (5.0)	1,013 (5.5)	1.09 (0.93-1.27)
28,801~36,300	259 (5.7)	893 (4.9)	1.39 (1.19-1.62)
36,301~45,800	351 (7.7)	1,186 (6.5)	1.42 (1.24-1.63)
>45,800	221 (4.8)	1,193 (6.5)	0.88 (0.75-1.03)
Medication use before cancer diagnosis			
Insulin glargine/detemir	268 (5.9)	549 (3.0)	2.03 (1.75-2.37)
Oral antidiabetic drugs	3,982 (87.1)	16,738 (91.5)	0.62 (0.56-0.68)
ACEi/ARBs	248 (5.4)	1,204 (6.6)	0.81 (0.70-0.93)
Alpha-blockers	42 0.9)	146 (0.8)	1.16 (0.82-1.64)
Beta-blockers	579 (12.5)	1,541 (8.4)	1.57 (1.42-1.74)
Calcium channel blockers	212 (4.6)	823 (4.5)	1.03 (0.88-1.21)
Dihydropyridine	203 (4.4)	803 (4.4)	1.01 (0.86-1.19)
Non-dihydropyridine	9 (0.2)	23 (0.1)	1.57 (0.72-3.38)
Diuretics	342 (7.5)	424 (2.3)	3.44 (2.97-3.99)
Statins	420 (9.2)	3,266 (17.9)	0.45 (0.41-0.50)
Fibrates	298 (6.5)	2,268 (12.4)	0.48 (0.43-0.55)
Comorbidities			
Liver cirrhosis	2,503 (54.7)	1,030 (5.6)	20.4 (18.4-22.6)
Chronic hepatitis B	2,303 (50.4)	868 (4.8)	20.4 (18.3-22.7)

Chronic hepatitis C	1,409 (30.8)	236 (1.3)	34.5 (29.3-40.7)
Hypertension	1,666 (36.4)	6,877 (37.6)	0.95 (0.89-1.02)
Hyperlipidemia	441 (9.6)	3,348 (18.3)	0.48 (0.43-0.53)
Ischemic heart disease	337 (7.4)	1,545 (8.5)	0.86 (0.76-0.97)
Myocardial infarction	29 (0.6)	151 (0.8)	0.77 (0.52-1.14)
Heart failure	140 (3.1)	453 (2.5)	1.25 (1.03-1.51)
Atrial fibrillation	61 (1.3)	194 (1.1)	1.26 (0.94-1.69)
Cerebrovascular disease	280 (6.1)	1,462 (8.0)	0.75 (0.65-0.85)
Stroke	185 (4.1)	919 (5.0)	0.80 (0.68-0.94)
Peripheral vascular disease	25 (0.6)	80 (0.4)	1.25 (0.80-1.97)
Chronic kidney disease	128 (2.8)	355 (1.9)	1.46 (1.19-1.79)
Depression	79 (1.7)	288 (1.6)	1.10 (0.86-1.41)
Charlson index	2.5 (2.4)	1.0 (1.2)	1.62 (1.59-1.66)
Resource utilization			
Number of A1C measurement	4.6 (5.7)	4.2 (5.6)	1.01 (1.01-1.02)
Number of lipid measurement	3.5 (4.5)	3.8 (5.0)	0.98 (0.97-0.99)
Number of outpatient visits	73.5 (81.2)	63.5 (74.6)	1.00 (1.00-1.00)
Number of hospitalization	1.7 (2.7)	0.9 (2.0)	1.17 (1.15-1.19)
Length of hospital stay > 7 days	0.8 (1.6)	0.4 (1.3)	1.21 (1.18-1.24)

Data presented as mean (standard deviation) or number (percent).

ACEi, Angiotensin-converting-enzyme inhibitor; ARB, angiotensin II receptor blocker.

Table S3 Hepatocellular carcinoma cases and matched controls among newly diagnosed type 2 diabetes patients without chronic viral hepatitis prescribed any antidiabetic agents (from 2004 to 2013)

	Cases (N=952)	Controls (N=3,808)	Crude OR (95% CI)
Age	65.2 (11.47)	64.7 (11.51)	-
Male	724 (76.1)	2,896 (76.1)	-
Year of initiating antidiabetic agents			
2004	155 (16.3)	620 (16.3)	-
2005	167 (17.5)	668 (17.5)	-
2006	118 (12.4)	472 (12.4)	-
2007	144 (15.1)	576 (15.1)	-
2008	107 (11.2)	428 (11.2)	-
2009	96 (10.1)	384 (10.1)	-
2010	80 (8.4)	320 (8.4)	-
2011	49 (5.2)	196 (5.2)	-
2012	31 (3.3)	124 (3.3)	-
2013	5 (0.5)	20 (0.5)	
Socioeconomic status (monthly income in NTD)			
≤17,280	406 (42.7)	1,733 (45.5)	Reference
17,281~22,800	371 (39.0)	1,372 (36.0)	1.16 (0.99-1.36)
22,801~28,800	42 (4.4)	159 (4.2)	1.14 (0.79-1.64)
28,801~36,300	42 (4.4)	135 (3.6)	1.34 (0.93-1.94)
36,301~45,800	50 (5.3)	229 (6.0)	0.93 (0.67-1.30)
>45,800	41 (4.3)	180 (4.7)	0.97 (0.67-1.41)
Medication use before cancer diagnosis			
Insulin glargine/detemir	48 (5.0)	132 (3.5)	1.49 (1.06-2.10)
Oral antidiabetic drugs	804 (84.5)	3,498 (91.9)	0.48 (0.38-0.59)
ACEi/ARBs	55 (5.8)	292 (7.7)	0.73 (0.54-0.99)
Beta-blockers	104 (10.9)	321 (8.4)	1.35 (1.06-1.71)
Calcium channel blockers	51 (5.4)	197 (5.2)	1.04 (0.75-1.43)
Dihydropyridine	48 (5.0)	190 (5.0)	1.01 (0.73-1.41)
Non-dihydropyridine	3 (0.3)	8 (0.2)	1.50 (0.40-5.65)
Diuretics	66 (6.9)	108 (2.8)	2.59 (1.88-3.56)
Statins	112 (11.8)	675 (17.7)	0.61 (0.49-0.76)
Fibrates	79 (8.3)	455 (12.0)	0.66 (0.51-0.85)
Comorbidities			
Liver cirrhosis	228 (24.0)	20 (0.5)	64.1 (37.4-110)
Hypertension	423 (44.4)	1,519 (39.9)	1.21 (1.05-1.40)

Hyperlipidemia	111 (11.7)	667 (17.5)	0.61 (0.49-0.76)
Ischemic heart disease	104 (10.9)	353 (9.3)	1.21 (0.96-1.53)
Myocardial infarction	10 (1.1)	40 (1.1)	1.00 (0.50-2.01)
Heart failure	53 (5.6)	137 (3.6)	1.60 (1.15-2.22)
Atrial fibrillation	16 (1.7)	71 (1.9)	0.90 (0.52-1.56)
Cerebrovascular disease	91 (9.6)	382 (10.0)	0.95 (0.74-1.21)
Stroke	56 (5.9)	248 (6.5)	0.90 (0.66-1.21)
Peripheral vascular disease	6 (0.6)	20 (0.5)	1.20 (0.48-2.99)
Chronic kidney disease	42 (4.4)	80 (2.1)	2.17 (1.48-3.18)
Depression	10 (1.1)	75 (2.0)	0.52 (0.27-1.02)
Charlson index	2.5 (2.6)	1.0 (1.3)	1.58 (1.51-1.66)
Resource utilization			
Number of A1C measurement	4.3 (5.4)	4.3 (5.9)	1.00 (0.99-1.02)
Number of lipid measurement	3.7 (4.6)	3.9 (5.0)	0.99 (0.97-1.01)
Number of outpatient visits	73.0 (80.5)	67.9 (75.2)	1.00 (1.00-1.00)
Number of hospitalization	1.8 (2.6)	1.0 (2.2)	1.14 (1.11-1.18)
Length of hospital stay > 7 days	0.9 (1.6)	0.5 (1.5)	1.17 (1.12-1.23)

Data presented as mean (standard deviation) or number (percent).

ACEi, angiotensin-converting-enzyme inhibitor; ARB, angiotensin II receptor blocker.

Table S4 Risk of hepatocellular carcinoma associated with all insulin analogues among newly diagnosed type 2 diabetes patients prescribed any antidiabetic agents before and after exclusion of patients with chronic viral hepatitis

	After exclusion of patients with chronic viral hepatitis				Before exclusion of patients with chronic viral hepatitis			
	HCC cases	Controls	Crude OR	Adjusted OR†	HCC cases	Controls	Crude OR	Adjusted OR‡
Nonuse	1,125	4,711	1.0	1.0	5,243	22,140	1.0	1.0
Any use	112	237	1.98 (1.57-2.50)	1.30 (0.96-1.75)	589	1,188	2.11 (1.90-2.34)	1.20 (1.03-1.40)
Current use	68	127	2.24 (1.65-3.02)	1.32 (0.88-1.97)	396	692	2.44 (2.15-2.78)	1.28 (1.05-1.55)
Recent use	9	33	1.13 (0.54-2.37)	0.44 (0.17-1.19)	47	120	1.63 (1.16-2.28)	0.66 (0.41-1.06)
Past use	35	77	1.92 (1.28-2.88)	1.74 (1.08-2.80)	146	376	1.66 (1.37-2.02)	1.29 (0.99-1.68)
Cumulative dosage								
High	42	74	2.36 (1.60-3.47)	1.50 (0.92-2.44)	217	373	2.48 (2.09-2.94)	1.40 (1.08-1.81)
Intermediate	33	84	1.66 (1.10-2.50)	1.08 (0.64-1.82)	162	425	1.62 (1.35-1.95)	0.97 (0.75-1.26)
Low	37	79	1.95 (1.31-2.89)	1.32 (0.80-2.20)	210	390	2.30 (1.94-2.73)	1.27 (0.99-1.63)
Cumulative duration								
Long	38	79	2.01 (1.36-2.99)	1.39 (0.84-2.30)	199	393	2.17 (1.82-2.59)	1.22 (0.94-1.57)
Intermediate	39	76	2.14 (1.45-3.17)	1.44 (0.88-2.36)	197	396	2.10 (1.77-2.50)	1.14 (0.88-1.47)
Short	35	82	1.79 (1.19-2.68)	1.08 (0.64-1.81)	193	399	2.06 (1.73-2.45)	1.24 (0.97-1.60)

All insulin analogues included insulin glargine, insulin detemir and premixed insulin analogues (insulin aspart plus insulin aspart protamine and insulin lispro plus insulin lispro protamine).

†Adjusted for liver cirrhosis, hypertension, hyperlipidemia, cerebrovascular disease, Charlson score index, oral antidiabetic drugs and statins.

‡Adjusted for socioeconomic status, chronic hepatitis B, chronic hepatitis C, liver cirrhosis, hyperlipidemia, heart failure, cerebrovascular disease, chronic kidney disease, Charlson score index, oral antidiabetic drugs, diuretics, statins, fibrates, aspirin, number of A1C measurement, number of lipid measurement and number of outpatient visits.

HCC, hepatocellular carcinoma.

Table S5 Risk of hepatocellular carcinoma associated with insulin glargine or detemir among newly diagnosed type 2 diabetes patients prescribed any antidiabetic agents before and after exclusion of patients with chronic viral hepatitis (from 2004 to 2013)

	After exclusion of patients with chronic viral hepatitis				Before exclusion of patients with chronic viral hepatitis			
	HCC cases	Controls	Crude OR	Adjusted OR†	HCC cases	Controls	Crude OR	Adjusted OR‡
Nonuse	904	3,676	1.0	1.0	4,305	17,743	1	1.0
Any use	48	132	1.49 (1.06-2.10)	0.91 (0.58-1.42)	268	549	2.03 (1.75-2.37)	1.04 (0.84-1.30)
Current use	21	50	1.73 (1.03-2.92)	1.06 (0.54-2.07)	149	274	2.26 (1.84-2.77)	1.15 (0.86-1.55)
Recent use	6	23	1.07 (0.43-2.66)	0.57 (0.16-2.09)	27	70	1.60 (1.02-2.50)	0.82 (0.45-1.50)
Past use	21	59	1.45 (0.87-2.40)	0.88 (0.46-1.68)	92	205	1.88 (1.46-2.41)	0.97 (0.68-1.39)
Cumulative dosage								
High	16	43	1.53 (0.85-2.74)	1.11 (0.54-2.31)	85	187	1.88 (1.45-2.44)	1.02 (0.70-1.47)
Intermediate	14	47	1.22 (0.67-2.22)	0.63 (0.28-1.40)	75	197	1.60 (1.22-2.09)	0.77 (0.52-1.14)
Low	18	42	1.76 (1.01-3.08)	1.04 (0.49-2.18)	108	165	2.73 (2.13-3.49)	1.38 (0.97-1.97)
Cumulative duration								
Long	14	44	1.30 (0.70-2.41)	0.96 (0.44-2.08)	88	183	2.00 (1.54-2.58)	0.97 (0.67-1.40)
Intermediate	16	43	1.52 (0.85-2.72)	0.93 (0.44-1.93)	86	188	1.90 (1.47-2.46)	1.13 (0.79-1.62)
Short	18	45	1.64 (0.94-2.86)	0.85 (0.40-1.78)	94	178	2.22 (1.72-2.86)	1.03 (0.71-1.49)

†Adjusted for liver cirrhosis, hyperlipidemia, cerebrovascular disease, Charlson score index, oral antidiabetic drugs, statins and fibrates.

‡Adjusted for socioeconomic status, chronic hepatitis B, chronic hepatitis C, liver cirrhosis, hyperlipidemia, heart failure, cerebrovascular disease, chronic kidney disease, Charlson score index, oral antidiabetic drugs, diuretics, statins, fibrates, aspirin, number of A1C measurement, number of lipid measurement and number of outpatient visits.

HCC, hepatocellular carcinoma.

Table S6 Interaction between insulin glargine or detemir and chronic viral hepatitis on risk of hepatocellular carcinoma among newly diagnosed type 2 diabetes patients prescribed any antidiabetic agents

Insulin glargine/detemir	Chronic viral hepatitis	HCC cases	Controls	Crude OR	Adjusted OR†
Non-use	Yes	2,374	938	22.0 (20.1-24.1)	7.41 (5.09-10.8)
Any use	No	97	503	1.68 (1.34-2.10)	1.22 (0.95-1.57)
Any use	Yes	171	46	32.3 (23.3-44.9)	8.42 (7.58-9.35)

Reference group: participants who had neither CHBC nor any use of insulin glargine/detemir.

P value for multiplicative interaction = 0.0918.

†Adjusted for socioeconomic status, liver cirrhosis, hyperlipidemia, heart failure, cerebrovascular disease, chronic kidney disease, Charlson score index, oral antidiabetic drugs, diuretics, statins, fibrates, aspirin, number of A1C measurement, number of lipid measurement and number of outpatient visits.

HCC, hepatocellular carcinoma.
